# Appraisal of ChatGPT’s Aptitude for Medical Education: Comparative Analysis With Third-Year Medical Students in a Pulmonology Examination

**DOI:** 10.2196/52818

**Published:** 2024-07-23

**Authors:** Hela Cherif, Chirine Moussa, Abdel Mouhaymen Missaoui, Issam Salouage, Salma Mokaddem, Besma Dhahri

**Affiliations:** 1 Faculté de Médecine de Tunis Université de Tunis El Manar Tunis Tunisia

**Keywords:** medical education, ChatGPT, GPT, artificial intelligence, natural language processing, NLP, pulmonary medicine, pulmonary, lung, lungs, respiratory, respiration, pneumology, comparative analysis, large language models, LLMs, LLM, language model, generative AI, generative artificial intelligence, generative, exams, exam, examinations, examination

## Abstract

**Background:**

The rapid evolution of ChatGPT has generated substantial interest and led to extensive discussions in both public and academic domains, particularly in the context of medical education.

**Objective:**

This study aimed to evaluate ChatGPT’s performance in a pulmonology examination through a comparative analysis with that of third-year medical students.

**Methods:**

In this cross-sectional study, we conducted a comparative analysis with 2 distinct groups. The first group comprised 244 third-year medical students who had previously taken our institution’s 2020 pulmonology examination, which was conducted in French. The second group involved ChatGPT-3.5 in 2 separate sets of conversations: without contextualization (V1) and with contextualization (V2). In both V1 and V2, ChatGPT received the same set of questions administered to the students.

**Results:**

V1 demonstrated exceptional proficiency in radiology, microbiology, and thoracic surgery, surpassing the majority of medical students in these domains. However, it faced challenges in pathology, pharmacology, and clinical pneumology. In contrast, V2 consistently delivered more accurate responses across various question categories, regardless of the specialization. ChatGPT exhibited suboptimal performance in multiple choice questions compared to medical students. V2 excelled in responding to structured open-ended questions. Both ChatGPT conversations, particularly V2, outperformed students in addressing questions of low and intermediate difficulty. Interestingly, students showcased enhanced proficiency when confronted with highly challenging questions. V1 fell short of passing the examination. Conversely, V2 successfully achieved examination success, outperforming 139 (62.1%) medical students.

**Conclusions:**

While ChatGPT has access to a comprehensive web-based data set, its performance closely mirrors that of an average medical student. Outcomes are influenced by question format, item complexity, and contextual nuances. The model faces challenges in medical contexts requiring information synthesis, advanced analytical aptitude, and clinical judgment, as well as in non-English language assessments and when confronted with data outside mainstream internet sources.

## Introduction

Artificial intelligence (AI) has emerged as a transformative force across various aspects of modern life. Within the realm of AI, natural language processing (NLP) has gained significant attention as it involves the use of devices to replicate human cognitive processes, encompassing learning, problem-solving, and practical application [1,2]. An exemplary NLP model is ChatGPT, developed by OpenAI. This model uses deep learning algorithms trained on extensive data sets to generate responses simulating human-like interactions. This versatile dialogic agent holds promise in diverse applications, including customer service and chatbots [3,4].

Launched on November 30, 2022, ChatGPT quickly gained popularity, attracting a million users within its first week and achieving unprecedented growth. In June 2023 alone, the ChatGPT website received 1.66 billion visits, underscoring its widespread appeal and use [5,6].

While this rapid development of ChatGPT has generated both excitement and concern across various fields, the impact on medical education has been particularly intriguing [7]. This chatbot technology may present opportunities to revolutionize medical education, offering enhanced efficiency, interactivity, and realism in training scenarios [8,9]. However, these benefits come with significant challenges and uncertainties that need to be carefully addressed and navigated [10,11].

A paramount examination in the medical school curriculum is the pneumology examination. This pivotal assessment evaluates the comprehensive understanding of respiratory diseases and their management—a core competency for any medical practitioner.

Our study aims to evaluate the performance of ChatGPT in the context of pneumology examinations through a comparative analysis with that of third-year medical students.

## Methods

### Study Design and Participants

This research adopts a cross-sectional design and was conducted at the pneumology teaching section of the Faculty of Medicine of Tunis (FMT), Tunisia, in June 2023. The study uses a comparative approach, involving 2 distinct groups: ChatGPT and medical students.

The first group comprises 244 third-year medical students registered at the FMT. These students had previously taken the pulmonology examination in January 2020. The second group consists of ChatGPT-3.5, a freely available version of ChatGPT, which undertook the same pneumology examination in June 2023.

### Pneumology Examination

#### Question Selection

The pneumology examination of FMT of 2020 is a 90-minute test comprising 50 questions, written in French. These questions underwent validation within the pneumology section of FMT to cover a diverse range of knowledge levels, including both fundamental and advanced concepts. The examination assesses candidates’ competency in various fields of pneumology, such as clinical pneumology, microbiology, respiratory radiology, pharmacology, pathology, and thoracic surgery.

The administered version of the examination involved only 45 text-based questions to align with ChatGPT’s processing capabilities. Thus, 5 questions based on visual components (images, graphs, and illustrations) were excluded since ChatGPT lacks the ability to process this material within its conversational scope.

A comprehensive mapping of assessment parameters for the administered pneumology examination is presented in Table 1. It encompasses a total of 9 multiple choice questions (MCQs), 13 short open-ended questions (SOEQs), and 7 clinical scenarios. Among the clinical scenarios, 2 were structured with MCQs, while the remaining 5 were constructed with SOEQs.

**Table 1:** Assessment parameters and question distribution in pneumology examination.

Mapping of pulmonology examination		Findings
**Parameters**		
	Academic year	2020
	Target examinees	Third-year medical students
	Timing	90 minutes
	Grading scale	0-100
	Questions, n	45
**Question topics, n (%)**		
	Clinical pneumology	27 (60)
	Radiology	7 (16)
	Pharmacology	5 (11)
	Pathology	3 (7)
	Microbiology	2 (4)
	Thoracic surgery	1 (2)
**Question formats, n (%)**		
	Independent MCQs^a^	9 (20)
	Independent SOEQs^b^	13 (29)
	MCQ-structured clinical cases	7 (16)
	SOEQ-structured clinical cases	16 (35)
**Distribution by difficulty index, n (%)**		
	Low difficulty index items	12 (27)
	Intermediate difficulty index items	25 (56)
	High difficulty index items	8 (18)
**Distribution by discrimination index, n (%)**		
	Low discrimination index items	21 (47)
	Intermediate discrimination index items	13 (29)
	High discrimination index items	11 (24)

^a^MCQ: multiple choice question.

^b^SOEQ: short answer open-ended question.

#### Item Performance Indexes

Item performance indexes are crucial statistical measures used to assess the effectiveness and quality of test questions, ensuring the reliability and validity of the assessment. These indexes provide valuable insights into the performance of each item concerning difficulty level, discrimination, and its ability to differentiate between high- and low-performing students. In this study, we used common item performance indexes, including the difficulty index (D1) and the discrimination index (D2) [12,13].

The D1 represents the proportion of students who answered an item correctly, calculated by dividing the number of correct responses by the total number of students attempting the item. While the optimal item difficulty may vary based on the specific test format and intended learning outcomes, a value within the 0.3 to 0.7 range is generally preferred [14,15].

On the other hand, the D2 measures an item’s capability to differentiate between high-performing and low-performing students. It is determined by comparing the performance of students who achieved high scores on the overall test with those who scored low on the same test for a particular item. D2 levels are classified as follows: high discrimination (D2>0.7), intermediate discrimination (D2 values between 0.3 and 0.7), and low discrimination (D2<0.3) [14,15].

### Data Collection and Score System

The database, containing the results and scores of medical students who took the pneumology examination in 2020, along with corresponding performance indexes, was accessible in the pneumology section and used in our comparative analysis.

**Figure 1:**
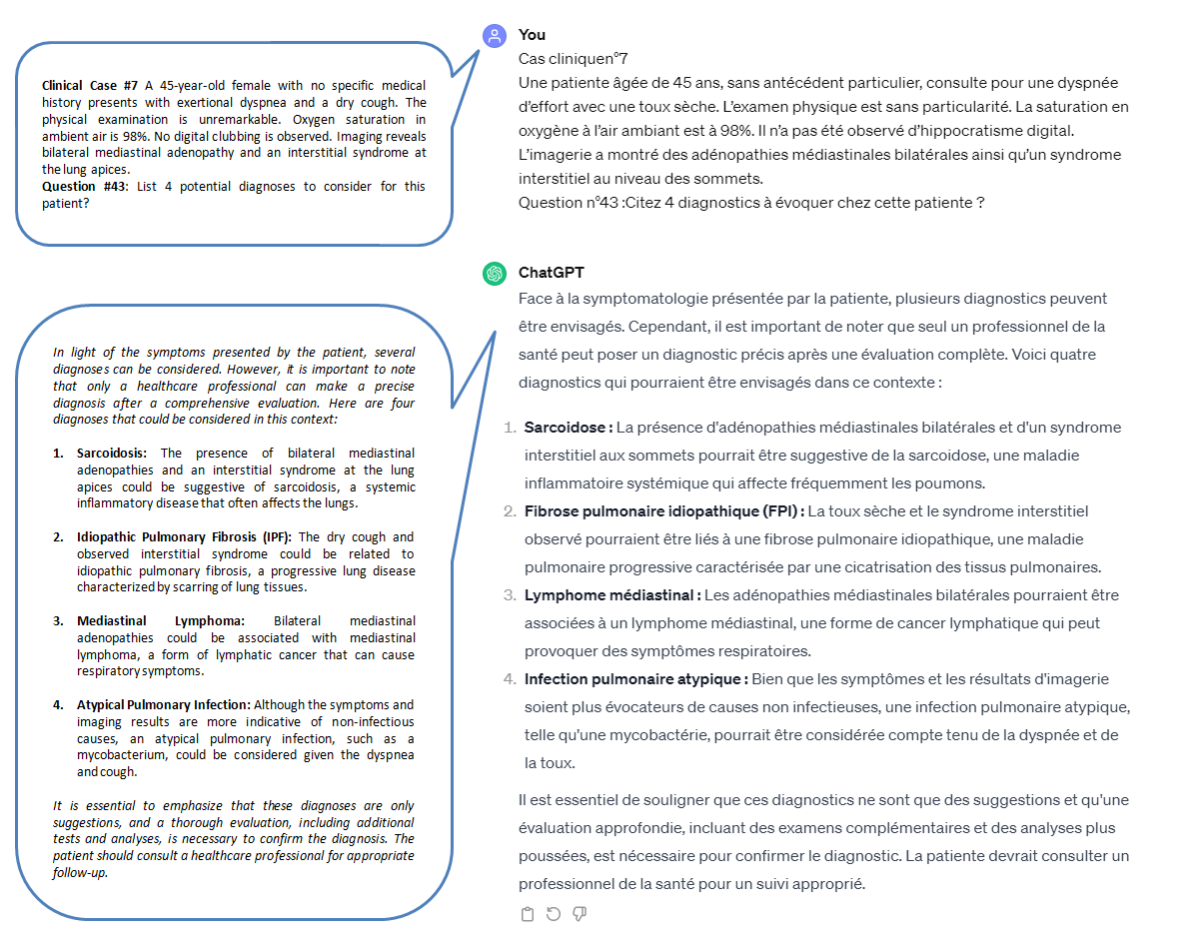
Illustration of the first version of the conversation with ChatGPT. The question was directly posed to ChatGPT without any context. ChatGPT responded by introducing the answer, emphasizing the necessity for a health care provider to address the case, and provide 4 probable diagnoses as demanded: sarcoidosis, idiopathic pulmonary fibrosis, mediastinal lymphoma, and atypical pulmonary infection. The bubbles in the figure represent the English translation of the conversations conducted in French.

**Figure 2:**
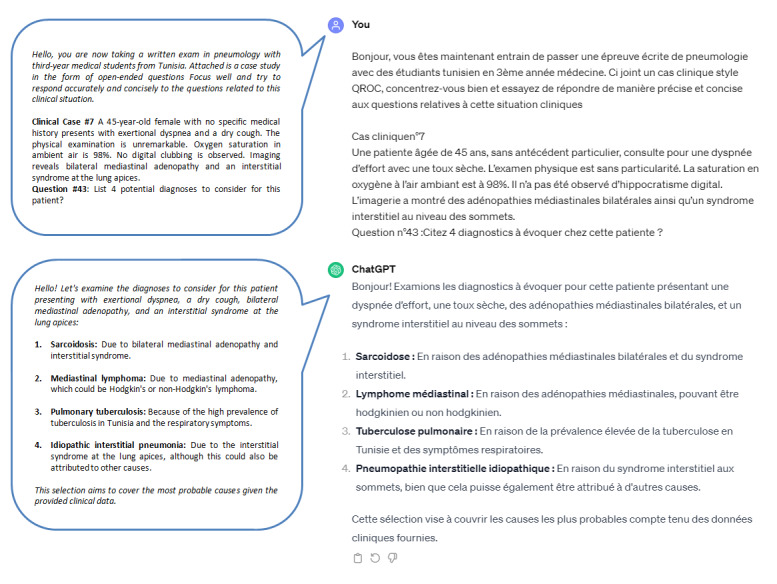
Illustration of the second version of the conversation with ChatGPT. The chatbot was asked the same question (in a separate chat session) but preceded by a briefing about the context of the question, which pertains to a pneumology examination for Tunisian medical students. A clear instruction on how to answer was provided. In light of this context, ChatGPT altered its response, explicitly stating pulmonary tuberculosis, likely due to the endemic nature of *Mycobacterium tuberculosis* in Tunisia and other African countries. The bubbles in the figure represent the English translation of the conversations conducted in French.

Two authors (HC and CM) conducted separate conversations with ChatGPT-3.5. In the first conversation, CM presented questions to the chatbot without contextualization (V1). In the second conversation, conducted by HC, suitable context was provided before posing the questions (V2). The questions were presented in exactly the same order as given to the students. Figures 1 and 2 show illustrations of the dual chat conversations conducted by HC and CM and the respective responses from ChatGPT.

The responses generated by both V1 and V2 were meticulously transcribed and stored in separate files. To ensure objectivity and independence, an impartial pneumology teacher, not involved in this study, conducted the evaluation. This teacher used the same grading scale specifically designed for evaluating student performance in the 2020 examination, ensuring an unbiased and rigorous assessment process.

Each question is assigned 1 point. For MCQs, the grading scale was as follows: an incorrect response concealed a correct answer. The assigned grades were 0, 1, or 0.5, based on the nature of the answer provided. SOEQs were assessed as follows: 1 point is awarded for a correct response, 0 points for an incorrect response, and 0.5 points for an omission. For clarity, the global scores achieved by both third-year medical students, and ChatGPT were transformed into a score out of 100 (maximum score). To successfully pass the examination, candidates needed to achieve a global score of ≥50 points.

### Data Analysis

The collected data underwent statistical analysis using SPSS (version 25.0; IBM Corp). Nominal values were expressed as frequencies and percentages, while continuous variables were represented using means and SDs for normally distributed data and medians and quartiles for non-normally distributed data.

A comparative analysis was conducted, evaluating student scores alongside those V1 and V2. This analysis encompassed various factors, including question formats (MCQs, SOEQs, and clinical scenarios), topics (clinical pneumology, microbiology, respiratory radiology, pharmacology, pathology, and thoracic surgery), and item performance indexes. To accurately portray the performance levels of each ChatGPT conversation, we presented results as percentages of the maximum scale attributed to each studied item, along with the ranking of ChatGPT scores among those of third-year medical students.

### Ethical Considerations

We have obtained approval from both the Medical Education Committee and the Ethics Committee of the Faculty of Medicine of Tunis to access the data (file number CE-FMT/2024/04/FSI/V2). This approval ensures confidentiality and restricting external use.

## Results

### Performance of Students in the Pneumology Examination

The median overall score achieved by medical students in the pulmonology examination was 48.9 out of 100 (IQR 40.0-54.7; Table 2). Among the participants (N=244), a modest cohort of 107 students reached the necessary threshold for successful completion of the examination, resulting in an overall success rate of 43.9%.

**Table 2:** Pneumology examination performance comparison: medical students versus ChatGPT with (V1) and without (V2) contextualization.

Parameters and categories		Maximum category score	Medical students’ performance	V1 performance					V2 performance		
			Score, median (IQR)	Score		Percentage score	Rank among students (percentile)	Score		Percentage score	Rank among students (percentile)
**Examination topics**											
	Pathology	3	2.5 (2-3)	2	66.7		133 (40.6)	2.5		83.3	84 (62.5)
	Pharmacology	5	3.5 (2.5-4)	3	60		137 (38.8)	3.5		70	96 (57.1)
	Microbiology	2	1.5 (1-1.5)	1.5	75		48 (78.6)	1.5		75	48 78.6)
	Radiology	7	3.5 (2.1-4.5)	3.5	50		93 (58.5)	4		57.1	64 (71.4)
	Thoracic surgery	1	0 (0-0)	1	100		1 (99.6)	0		0	29 (87.1)
	Clinical pneumology	27	11 (9-13)	10	37		133 (40.6)	11.5		42.6	97 (56.7)
**Question formats**											
	Independent MCQs^a^	9	4.5 (3.5-5.5)	4	44.4		138 (38.4)	3		33.3	191 (14.7)
	Independent SOEQs^b^	13	5 (3.5-6)	4.5	34.6		120 (46.4)	6.5		50	30 (86.6)
	MCQ-structured clinical cases	7	2.8 (2-3.5)	1.5	21.4		181 (19.2)	2		28.6	149 (33.5)
	SOEQ-structured clinical cases	16	9.5 (7.6-11)	11	68.8		51 (77.2)	11.5		71.9	36 (83.9)
Overall examination score		100	48.9 (40-54.4)	46.7	46.7		133 (40.6)	51.1		51.1	85 (62.1)

^a^MCQ: multiple choice question.

^b^SOEQ: short answer open-ended question.

Significant variations in performance emerged across different question categories. Notably, students (N=244) demonstrated pronounced proficiency in the domains of pathology, pharmacology, and microbiology, with scores exceeding 50% in 88.5% (n=216), 77.5% (n=189), and 74.6% (n=182), respectively. A moderate level of accomplishment was observed in the field of radiology. In contrast, the weakest performances were evident in questions related to thoracic surgery and clinical pneumology, with only 11.5% (n=28) and 22.5% (n=55) of students surpassing the 50% threshold of the maximum score in these areas.

The question format also appeared to significantly influence students’ performance. Candidates (N=244) excelled in SOEQ-structured clinical cases and independent MCQs, with 68.9% (n=212) and 56.1% (n=137), respectively, achieving marks exceeding 50% of the maximum achievable. Conversely, the performance in MCQ-structured clinical cases lagged, with only 31.1% (n=76) of candidates reaching scores beyond 50% of the highest attainable mark for this question format. The most challenging performance was observed in independent SOEQs, as only 19.3% (n=47) of students achieved marks surpassing the 50% threshold of the maximum attainable for this particular question format.

Based on these students’ outcomes, item performance indexes were computed. A significant proportion of questions (25/45, 56%) exhibited moderate difficulty indexes, while only 18% (8/45) of the questions demonstrated elevated levels of difficulty. Additionally, a substantial fraction of the items (21/45, 47%) showed limited discriminatory power in contrast to 24% (11/45) that displayed a pronounced D2 (Table 1).

### Assessment of ChatGPT-3.5 Performance in the Pneumology Examination

V1 performed well, achieving scores exceeding 50% in all question categories except for clinical pneumology. A similar trend emerged with V2, even though it faced challenges in reaching scores above 50% in thoracic surgery and clinical pneumology (Table 2).

The question format significantly impacted ChatGPT’s performance. In cases where questions lacked contextualization, V1 fell short of reaching the 50% mark for the maximum score in all question formats, except for SOEQ-structured clinical cases. Similarly, in the responses generated by V2, even when provided with appropriate context, limitations were evident in both independent MCQs and MCQs integrated into clinical cases. Interestingly, V2 demonstrated a higher level of accuracy in SOEQ-structured clinical cases. Both conversations displayed improved performance in questions with higher D1 and D2 (Table 3).

Considering the overall examination scores, V1 did not meet the passing threshold, achieving a total score of 46.7 out of 100. In contrast, V2 secured a global score of 51.5 out of 100, narrowly achieving success in this examination.

**Table 3:** Achievement quotient of ChatGPT with (V1) and without (V2) contextualization in the pneumology examination by difficulty and discrimination indexes.

	V1 (%)	V2 (%)
Low difficulty index terms	20.8	16.7
Intermediate difficulty index terms	54	62
High difficulty index terms	62.5	68
Low discrimination index items	31	38.1
Intermediate discrimination index items	61.5	61.5
High discrimination index items	59.1	63.6

### Comparative Analysis of ChatGPT Performance and Medical Students’ Performance

#### Question Topic

Comparing the performance of ChatGPT with that of medical students, distinct patterns emerge. V1 demonstrated heightened proficiency in specialized pneumology fields, especially radiology, microbiology, and thoracic surgery. Notably, V1 outperformed 131 (58.5%), 176 (78.6%), and 223 (99.6%) medical students in these respective domains. ChatGPT faced challenges in this conversation when addressing questions related to pathology, pharmacology, and clinical pneumology, achieving lower scores than most medical students. In opposition to that, V2 consistently provided more accurate responses than the majority of medical students across various question categories, regardless of their specialized fields. Noteworthy excellence was observed, particularly in microbiology and thoracic surgery.

#### Question Format

V1 demonstrated strong proficiency in SOEQ-structured clinical cases, surpassing the performance of 173 (77.2%) medical students. However, its performance weakened in independent MCQs and SOEQs, and it performed less optimally in MCQ-structured clinical cases compared to third-year medical students. In the case of V2, commendable performance was observed in responding to both independent and structured SOEQs within clinical cases. Yet, a notable deficiency emerged in accurately answering all formats of MCQs, ranking only above 33 (14.7%) and 75 (33.5%) students in independent MCQs and MCQ-structured clinical cases, respectively.

#### Item Performance Indexes

**Figure 3:**
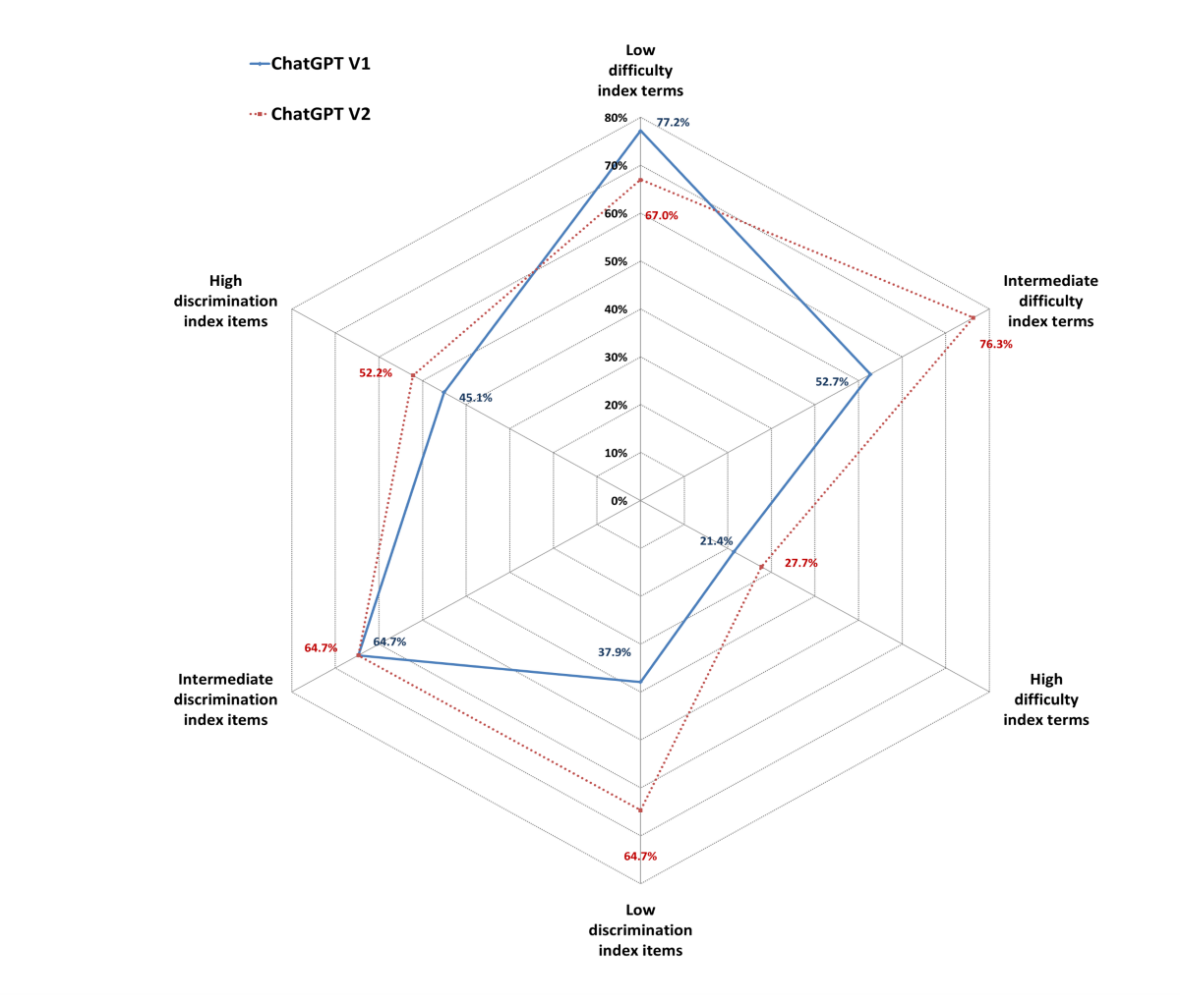
Percentile rank of ChatGPT with (ChatGPT-V1) and without (ChatGPT-V2) contextualization among medical students in the pneumology examination based on difficulty and discrimination indexes. Percentages represent the percentile rank of ChatGPT-V1 and ChatGPT-V2 among medical students.

Both conversations with ChatGPT, particularly V2, performed better than students in handling questions of low and intermediate difficulty. Remarkably, students demonstrated stronger proficiency when tackling highly difficult questions. Regarding the D2, V1 showed similar performance to participants in accurately addressing questions with low and high D2 index values. Additionally, V1 slightly exceeded participants’ performance in questions with an intermediate D2 index. V2 consistently outperformed medical students across all question discrimination categories (Figure 3).

In summary, V1 did not pass the examination, but its score surpassed that of 91 (40.6%) students. In contrast, V2 successfully passed the examination, outperforming 139 (62.1%) medical students.

## Discussion

### Principal Findings

The cognitive capabilities and knowledge processing of ChatGPT have generated significant discussions in both public and academic circles. This NLP tool has gained attention for its prompt and coherent responses across various subjects, showcasing an impressive capacity to generate essays and offer explanations. However, there is a lack of comprehensive investigations into ChatGPT’s performance in medical education and examinations. To address this, this study evaluates ChatGPT using a previously collected data set of pneumology examinations from FMT, enabling direct comparisons between ChatGPT’s performance and that of third-year medical students.

Our findings highlight ChatGPT’s proficiency in handling diverse biomedical information and clinical data. Powered by a vast corpus of internet text data, ChatGPT demonstrates remarkable expertise in pneumology, particularly excelling in radiology and microbiology. It outperformed a significant proportion of medical students in these paraclinical specialties. Comparable high performance of AI-powered tools in paraclinical sciences has been previously documented before. Rodriguez-Ruiz et al [16], using data from 9 diverse data sets (2652 examinations), including 653 malignancies, found that their AI system exhibited cancer detection accuracy on par with the average breast radiologist, surpassing the performance of 61.4% of the radiologists in their retrospective analysis.

Das et al [17] assessed ChatGPT’s accuracy in addressing a test based on the competency-based medical education (CBME) curriculum for microbiology. ChatGPT showcased the ability to answer both first- and second-order knowledge questions related to microbiology. The model exhibited significant potential as an automated question-answering tool in the field of microbiology, achieving an accuracy rate of approximately 80%. In another investigation, ChatGPT demonstrated proficiency in medical biochemistry, another paraclinical specialty. It successfully responded to 200 random medical biochemistry reasoning questions from the CBME curriculum’s competency modules [8].

In fields like clinical pneumology that demand careful processing of medical data, ChatGPT shows some limitations when compared to medical students. However, these shortcomings can be improved through adequate contextualization, as seen in the enhanced proficiency of V2. Our findings about clinical pneumology align with previous studies that highlight ChatGPT’s challenges in similar medical disciplines requiring advanced judgment and nuanced clinical reasoning, such as neurology and traumatology. For instance, ChatGPT 3.5 achieved an overall accuracy rate of 57%, just below the 58% passing threshold set for the 2022 UK Specialty Certificate Neurology Examination [18].

Moreover, ChatGPT scored 35.8%, which is notably lower than the pass rate for the Fellowship of the Royal College of Surgeons examination in trauma surgery by 30%. This performance was also 8.2% below the average score of participants at all training levels [19]. In a study conducted in India, ChatGPT demonstrated a limited ability to translate basic pharmacology knowledge into clear clinical concepts. It exhibited inconsistency in predicting and explaining common drug interactions [20]. This observation aligns with ChatGPT’s modest accuracy in questions related to pharmacology applied to pneumology in our FMT examination.

The way questions are presented greatly affects how well both medical students and AI tools like ChatGPT perform [21,22]. ChatGPT struggled to match the performance of medical students in all question styles, except for SOEQs integrated into clinical scenarios. Even after contextualization, ChatGPT still had a hard time answering MCQs in pulmonology compared to medical students. Zhu et al [23] addressed this concern, suggesting that ChatGPT may be more suitable for responding to open-ended questions than for being presented with a predefined set of options. Considering the ChatGPT’s occasional inconsistency in providing identical responses for the same question, the authors recommended posing the question 3 times to ensure response stability.

Other research generally shows good performance by ChatGPT when handling MCQs. For example, a 2023 study by Duong and Solomon [24] revealed ChatGPT’s comparable performance to human beings in responding to MCQs on human genetics. ChatGPT also successfully passed the 2022 Italian Residency Admission National Exam, which consists solely of MCQs. Additionally, in the 2022 European Examination in Core Cardiology, ChatGPT answered over 60% of questions correctly, displaying consistency across various MCQs [25]. In this study, the discrepancy in ChatGPT’s performance across question formats may be attributed to the high difficulty level of these questions, even for third-year medical students.

ChatGPT clearly outperformed medical students in tasks that required detailed responses, particularly SOEQs integrated into clinical scenarios. This was supported by Qu et al [26], who also emphasized the impressive capability of this NLP software in handling otorhinolaryngology clinical scenarios [26]. Indeed, ChatGPT consistently provided accurate differential diagnoses and well-justified treatment strategies for recognized clinical conditions. It used specialized medical terminology and carefully curated relevant medical history, physical examination, radiological, and laboratory findings. This proficiency can be explained by the similarity between the scenarios in our pneumology examination and the writing style commonly found in textbooks, scientific literature, and other data sources used to train the AI model.

Unlike third-year medical students, ChatGPT surprisingly exhibited limited performance on questions with a high difficulty index. These questions necessitate skills in navigating intricate concepts, synthesizing information, and using strategic analytical abilities. Bhayana et al [27] subjected this chatbot to the Canadian Royal College and American Board of Radiology examinations and their conclusions match our findings. Although ChatGPT successfully passed these examinations, it faced difficulties with questions demanding higher order thinking, such as describing radiological findings, classification, and application of concepts [27]. While certain questions can help tell the difference between students with different levels of ability or knowledge, this D2 might not apply directly to AI-powered models like ChatGPT. A noteworthy observation is ChatGPT’s enhanced performance when provided with adequate context, outperforming students irrespective of the theoretical item discrimination.

Ultimately, the findings reveal unexpected limits in ChatGPT’s performance during our pneumology examination. It barely passed in the part with contextualized chats, giving an overall modest score of 51.1%. This is different from past research where ChatGPT consistently demonstrates strong performance in English-language medical assessments like the United States Medical Licensing Examination, CBME evaluations, and the European Examination in Core Cardiology [17,25,28]. It appears that its effectiveness diminishes when dealing with evaluations from non-Western institutions and non-English language examinations like our Tunisian examination, written in French. Similarly, this AI chatbot faced challenges in both the Taiwanese pharmacist licensing and Taiwanese family medicine board examinations [29,30]. It also scored below the level of students in a Korean parasitology examination, the Japanese National Medical Licensing Examination, and the Chinese National Medical Licensing Examination [31,32]. This discrepancy likely arises from ChatGPT’s limited ability to grasp linguistic nuances in non-English texts, exacerbated by the prevalence of Western-centric internet data. In certain contexts, these data may not fully apply to African and Asian populations, which exhibit slight variations in clinical presentations and disease epidemiology.

### Strengths and Limitations of the Study

Our research constitutes the initial exploration of ChatGPT’s capabilities in French-language medical examinations, providing a valuable addition to the expanding body of research in medical AI assessment. A notable strength of this study lies in its comparative approach, effectively evaluating ChatGPT’s performance alongside that of medical students in a comprehensive pneumology examination. This examination covers various question formats and topics, offering a realistic assessment of the AI’s competencies.

However, the study acknowledges several limitations. Conducted at a single institution with a highly homogeneous population concerning demographics, educational background, and medical curricula, there may be a potential selection bias that affects the external validity of the findings, particularly when extrapolating to more diverse student groups, even from other French-speaking medical universities. Additionally, focusing solely on the pneumology field may limit the generalizability of the findings to a broader academic context.

ChatGPT’s inability to process visual elements also introduces an inherent selection bias concerning the administered questions, hindering a comprehensive evaluation of its proficiency in clinical scenarios where visual cues, radiology data, and histological images are significant. It is crucial to recognize that the specific findings related to ChatGPT-3.5 may not necessarily extend to other iterations of ChatGPT or alternative AI models. Furthermore, the absence of cultural adaptation and the scarcity of relevant data for non-Western contexts impeded a thorough exploration of ChatGPT’s capabilities, potentially introducing a cultural bias.

### Conclusions

In summary, despite its access to a comprehensive web-based data set and quick response generation, ChatGPT performs similarly to an average medical student, with outcomes influenced by question format, item complexity, and contextual factors. Notably, ChatGPT struggles in specific medical contexts requiring information synthesis, advanced analytical skills, and nuanced clinical judgment. Its efficiency also diminishes in non–English language assessments and when confronted with data outside dominant internet sources. These findings suggest the need for further exploration and improvement in the application of AI tools like ChatGPT in medical education, training, and evaluation. It also emphasizes the importance of enhancing its performance across cultural and linguistic contexts.

## Abbreviations

AI: artificial intelligence
CBME: competency-based medical education
D1: difficulty index
D2: discrimination index
FMT: Faculty of Medicine of Tunis
MCQ: multiple choice question
NLP: natural language processing
SOEQ: short open-ended question

## Data Availability

The data sets generated and analyzed during this study are available from the corresponding author on reasonable request.
